# X Marks the Clot: Evolutionary and Clinical Implications of Divergences in Procoagulant Australian Elapid Snake Venoms

**DOI:** 10.3390/toxins17080417

**Published:** 2025-08-18

**Authors:** Holly Morecroft, Christina N. Zdenek, Abhinandan Chowdhury, Nathan Dunstan, Chris Hay, Bryan G. Fry

**Affiliations:** 1 Adaptive Biotoxicology Lab, School of the Environment, University of Queensland, St Lucia, QLD 4072, Australia; h.morecroft@uq.net.au (H.M.); abhichy.official@gmail.com (A.C.); 2 Australian Reptile Academy, Ripley, QLD 4306, Australia; christinazdenek@gmail.com (C.N.Z.); tropidechis@hotmail.com (C.H.); 3 Venom Supplies, Tanunda, SA 5352, Australia; nathan@venomsupplies.com

**Keywords:** venom, coagulopathy, factor activation, antivenom, evolution

## Abstract

Australian elapid snakes possess potent procoagulant venoms, capable of inducing severe venom-induced consumption coagulopathy (VICC) in snakebite victims through rapid activation of the coagulation cascade by converting the FVII and prothrombin zymogens into their active forms. These venoms fall into two mechanistic categories: FXa-only venoms, which hijack host factor Va, and FXa:FVa venoms, containing a complete venom-derived prothrombinase complex. While previous studies have largely focused on human plasma, the ecological and evolutionary drivers behind prey-selective venom efficacy remain understudied. Here, thromboelastography was employed to comparatively evaluate venom coagulotoxicity across prey classes (amphibian, avian, rodent) and human plasma, using a taxonomically diverse selection of Australian snakes. The amphibian-specialist species *Pseudechis porphyriacus* (Red-Bellied Black Snake) exhibited significantly slower effects on rodent plasma, suggesting evolutionary refinement towards ectothermic prey. In contrast, venoms from dietary generalists retained broad efficacy across all prey types. Intriguingly, notable divergence was observed within *Pseudonaja textilis* (Eastern Brown Snake): Queensland populations of this species, and all other *Pseudonaja* (brown snake) species, formed rapid but weak clots in prey and human plasma. However, the South Australian populations of *P. textilis* produced strong, stable clots across prey plasmas and in human plasma. This is a trait shared with *Oxyuranus* species (taipans) and therefore represents an evolutionary reversion towards the prothrombinase phenotype present in the *Oxyuranus* and *Pseudonaja* last common ancestor. Clinically, this distinction has implications for the pathophysiology of human envenomation, potentially influencing clinical progression, including variations in clinical coagulopathy tests, and antivenom effectiveness. Thus, this study provides critical insight into the ecological selection pressures shaping venom function, highlights intraspecific venom variation linked to geographic and phylogenetic divergence, and underscores the importance of prey-focused research for both evolutionary toxinology and improved clinical management of snakebite.

## 1. Introduction

Venomous snakes have evolved a remarkable arsenal of toxins that disrupt physiological systems in prey, and Australian species are among the most notorious for their potent coagulotoxic effects on blood clotting [1,2,3,4,5,6,7,8,9,10,11,12,13,14,15,16,17,18,19,20,21,22,23,24,25,26,27,28,29,30,31,32,33,34,35,36,37,38,39,40,41,42]. At the base of the Australian snake radiation, the blood clotting enzyme Factor Xa was recruited for use in the biochemical arsenal, with Factor Va later added in the last common ancestor of *Oxyuranus* species (taipans) and *Pseudonaja* species (brown snakes) [43,44,45,46]. These toxins activate the clotting enzymes Factor VII and prothrombin [47]. They act as overdoses, triggering rampant out of control blood clotting, thereby consuming available clotting proteins. As such, envenomations by Australian elapids frequently cause venom-induced consumption coagulopathy (VICC) in human victims, a syndrome whereby procoagulant toxins convert clotting factor zymogens to their active enzymatic forms, which may result in thrombotic microangiopathy but ultimately leads to widespread clotting factor depletion resulting in incoagulable blood [3,15,16,22,29,30,33,48,49,50,51].

The presence of a pre-assembled Xa–Va complex in *Oxyuranus* and *Pseudonaja* venoms can greatly accelerate clotting; clinically, envenomation by *Pseudonaja* species (with a Group C prothrombin activator) triggers coagulopathy more rapidly than envenomation by species reliant on assembling host factor Va (such as species of *Cryptophis*, *Demansia*, *Hemiaspis*, *Hoplcephalus*, *Notechis*, *Suta*, *Tropidechis* and *Pseudechis porphyriacus* [22,45,47]. Thus, while all these venoms are highly toxic, their mechanisms of coagulation activation differ, with important consequences for the speed and nature of the coagulopathic effect in victims.

Although human snakebite is a serious medical issue, from the snake’s perspective venom is primarily an adaptation for subduing prey. Indeed, numerous studies have found a correlation between a snake’s typical prey and the toxicity profile of its venom [52,53,54,55,56,57,58,59,60,61,62,63,64,65,66,67,68,69,70,71,72,73,74,75]. Venoms tend to be most effective against the taxa that snakes commonly hunt, suggesting that natural selection tailors venom composition to prey physiology. This can lead to prey-specific venom actions, where the biochemical targets and potency of toxins align with particular prey classes [52,55,57,60,62,63,65,68,70,72,76,77,78,79,80,81,82,83,84,85,86,87,88,89]. Such diversification of venom function is thought to confer adaptive advantages, as it expands a predator’s ability to incapacitate various prey types or overcome prey-specific defenses. Conversely, species with very narrow diets may evolve venoms highly optimized to their preferred prey, potentially at the expense of efficacy in other animals. Empirical evidence supports this dichotomy: snakes with more generalized diets (feeding on a broad range of prey) often exhibit greater toxin diversity and a wider range of toxic effects in their venoms [60]. In contrast, dietary specialists can exhibit venom that is extremely potent to their target prey yet comparatively less effective in other taxa, reflecting an evolutionary trade-off in toxin specialization.

Despite this understanding, most venom research to date, particularly in the context of Australian elapids, has focused on human-relevant effects or standard laboratory models, rather than comparing venom action across different prey [45,46,90,91,92]. Coagulotoxic venom activity is typically characterized using human plasma or rodent assays to infer clinical risk, leaving a knowledge gap about how these same venoms act on the blood of animals that the snakes actually prey upon. Studying venom effects in diverse prey species is crucial, as it illuminates the evolutionary context of toxin function and may reveal selective refinements that are not apparent from human-focused research. Furthermore, variations in coagulotoxic action have direct implications for envenomation outcomes in people. Venom components that evolved to incapacitate amphibians or reptiles, for example, might induce a different pathological profile in humans than toxins adapted for killing mammals [57,70,75,88,93]. The diversity of clotting pathways targeted by various venom toxins means that human snakebite victims can experience complex coagulopathies when a venom contains multiple clot-activating mechanisms [94,95]. Understanding these nuances is important for clinical toxinology: it helps explain why envenomations by different species can produce somewhat different patterns of coagulopathy, and it underscores the need for appropriately designed antivenoms and medical interventions. In short, recognizing the prey-specific adaptations of venoms can improve our predictions of which coagulation factors will be affected in a given snakebite case, thereby informing treatment. For example, the extremely rapid VICC caused by *Pseudonaja* bites aligns with that species’ possession of a preformed prothrombinase complex (FXa in addition to FVa), whereas snakes with venom containing only FXa rely on activating host FVa produce coagulopathic symptoms with a slower timeline or severity [22,47].

In light of these considerations, the present study aimed to systematically examine the prey-selective coagulotoxic effects of Australian snake venoms and to relate these findings to potential impacts on human envenomation. We selected a taxonomically diverse panel of venoms from Australian elapid snakes and tested their procoagulant activity on the plasmas of three different model organisms that represent major prey categories, as well as on human plasma. Specifically, we used plasma from cane toad (an amphibian model organism), chicken (an avian model organism), and rat (a rodent model organism) to reflect common prey types, alongside human plasma to gauge clinical relevance. By measuring clotting dynamics (clotting time and clot strength via thromboelastography) induced by each venom, we determined how venom potency and clot quality vary across prey types in relation to human plasma effects. We hypothesized that venoms of specialist predators (e.g., snakes that predominantly feed on reptiles or amphibians) will show markedly lower coagulant efficacy in mammalian plasma compared to venoms of generalist feeders or those containing a complete prothrombinase (FXa:FVa) complex. Such a pattern would suggest that evolutionary pressures have fine-tuned those venoms for particular prey, resulting in diminished activity outside their target class. Conversely, we expected that generalist or broad-spectrum venoms maintain high clotting potency across multiple animal plasma types. Through this comparative approach, our study provides new insight into how prey selection has driven the evolution of coagulotoxic venom phenotypes. Importantly, the findings also form a foundation for understanding how these venom variations translate into differences in human coagulopathic response, thereby bridging ecological venom function with its medical consequences and revealing the limitations of using animal plasmas to predict human envenomation effects. In doing so, we hope to advance both the evolutionary biology of venom and the clinical management of snakebite envenomation.

## 2. Results

### 2.1. Animal Plasma Assays

**Figure 1:**
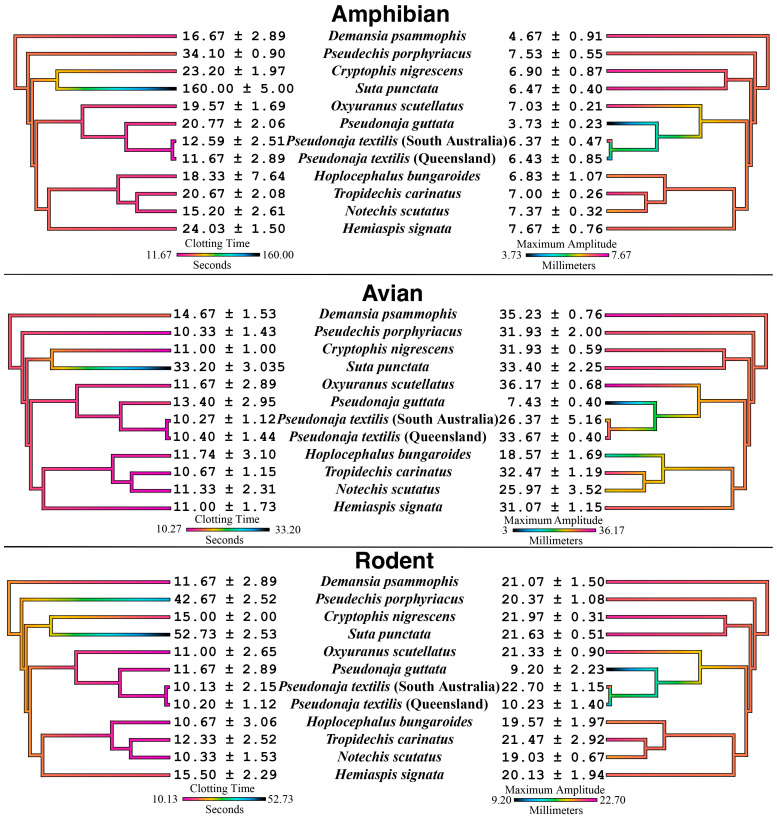
Australian elapid procoagulant effects upon animal plasmas mapped over the snake organismal phylogenetic tree comparing clotting time (R-values, the time taken until clot reaches 2 mm), and maximum amplitude, the maximum clot strength recorded (mm). Values are N = 3 mean plus standard deviation. The venom effects for each plasma type are mapped over the organismal tree, with the tree obtained from previously published genetic results [99,100]. Smaller clotting time values and warmer colors indicate more potent venoms. Conversely, larger maximum amplitude values and warmer colors indicate stronger clot formation.

The most notable variation was *P. porphyriacus* which was proportionally much slower acting relative in clotting rodent plasma than it proportionally was for amphibian and avian plasmas (Figure 1) (*p* < 0.0001). This species feeds primarily on amphibians or reptiles rather than on mammals [96]. Thus, the variations seen are suggestive of evolutionary selection pressures for selectivity for non-mammalian plasmas, with more precise binding to the coagulation proteins of amphibians and reptiles. In contrast, the other FXa-venoms, from species with more generalist diets, did not vary significantly between plasmas, thus being suggestive of more generalized acting venoms that have not been purified under selection pressures. Notable exceptions to this were the *Pseudonaja* venoms which produced some intriguing variations not in clotting time, but in the strength of the fibrin clot formed by the procoagulant venom actions (Figure 1). *Pseudonaja guttata* consistently produced the weakest clots despite being fast acting (except on amphibian plasma, where it was proportionally slower than its effect on avian and rodent plasma). *Pseudonaja textilis* (Queensland population) produced strong clots in amphibian and avian plasmas but produced weak clots in rodent plasma (*p* < 0.0001). In contrast, the South Australian population of *P. textilis* produced strong clots in all three animal plasmas.

### 2.2. Human Plasma Assays

**Figure 2:**
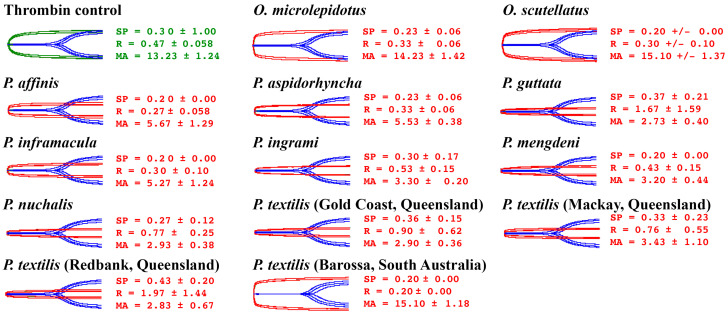
Thromboelastographic traces of the effects of the thrombin control (green), *Oxyuranus* (taipan), and *Pseudonaja* (brown snake) venoms (red traces) upon human plasma, overlayed over the spontaneous clotting control (blue). SP = Split point, time taken until a clot is formed (min). R = time taken until clot reaches 2 mm. MA = Maximum amplitude, indicating maximum clot strength (mm). Values are N = 3 mean plus standard deviation. The results demonstrate that, with the exception of the southern population of *Pseudonaja textilis* (shown by the Barossa, South Australian representative sample), *Pseudonaja* venoms uniquely produce weak, transient fibrin clots in contrast to the strong, stable clots produced by taipan (*Oxyuranus* species) venoms and that of other procoagulant Australian elapid snakes.

**Figure 3:**
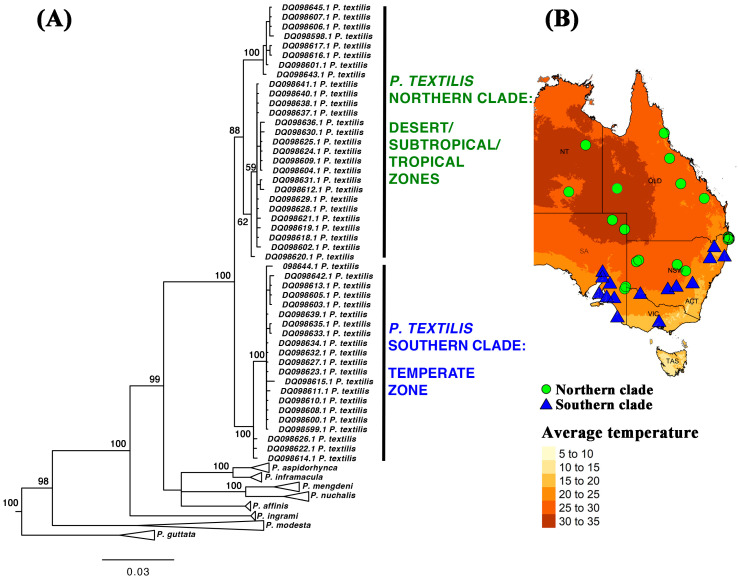
(**A**) *Pseudonaja* phylogenetic tree with a focus upon *P. textilis* (Eastern Brown Snake); results for other species are presented to show the relative position of *P. textilis* but are shown with the nodes collapsed. Tree is based upon 766 base-pair mitochondrial sequences (Genbank accession codes shown): NADH dehydrogenase subunit 4 (ND4) gene (partial cds); and tRNA-His and tRNA-Ser genes (complete sequences) [97]. Tree was rooted with *Oxyuranus* outgroup sequences. Alignment, MrBayes run file, and full tree output file are in Supplementary Files S1, S2, and S3, respectively. (**B**) Genetic results mapped over eastern Australia to reveal the ecozones occupied by each *P. textilis* clade. Localities linked to Genbank accession codes are in Supplementary File S4.

Factor Xa-only-containing venoms (species from genera other than *Oxyuranus* and *Pseudonaja*) had been previously tested for effects on human plasma, with all venoms producing strong stable clots [45]. Therefore, for these assays we focused the current study upon the effects of the FXa+FVa venoms from species in the *Oxyuranus* and *Pseudonaja* genera (Figure 2). All Oxyuranus venoms formed rapid clots with high maximum amplitudes (strong, stable clots). In contrast, *Pseudonaja* venoms also clotted very quickly but produced significantly lower maximum amplitudes, indicating weak, friable clots. Conspicuously, the southern population of *P. textilis* (Barossa, SA) produced strong fibrin clots (higher maximum amplitudes), effectively reversing the typical brown snake–weak clot pattern; being the only one not significantly different from *Oxyuranus* venoms (*p* > 0.9999 relative to both *Oxyuranus* species). However, the southern *P. textilis* (Barossa, SA, Australia) was significantly different from venoms from northern *P. textilis*: 0.0244 for *P. textilis* (Gold Coast, QLD, Australia); 0.0044 for *P. textilis* (Mackay, QLD, Australia); and 0.007 for *P. textilis* (Redbank, QLD, Australia), which all formed weak clots. It was also significantly different from the venoms of all other *Pseudonaja* species: *p* = 0.0133 for *P. affinis;* 0.0397 for *P. aspidorhyncha*; 0.0243 for *P. guttata;* 0.0062 for *P. mengdeni;* 0.0247 for *P. nuchalis.* The clot strength division within *P. textilis* is paralleled by a well-supported, deep genetic divide within this species. It is partitioned into two distinct clades: a northern clade from the high-temperature desert, subtropical, and tropical zones; and a southern clade from the temperate zone [97,98] (Figure 3). As *P. textilis* is not a basal species (Figure 3), this indicates that the weak clot trait evolved at the base of the *Pseudonaja* tree. Consequently, the ability of venom from the southern clade to form strong blood clots is a reversal back to the state present in the last common ancestor of *Oxyuranus* and *Pseudonaja*. 

## 3. Discussion

### 3.1. Prey-Driven Venom Specialization: Evolutionary Implications

Our findings support the paradigm that snake venom composition is shaped by dietary selection pressures, resulting in prey-specific toxin efficacy. Venoms from FXa-only amphibian- and reptile-specialist elapid species showed markedly increased coagulant speed of action towards amphibian or avian plasma relative to mammalian plasma. In contrast, venoms from more generalist feeders (and those in the *Oxyuranus/Pseudonaja* clade that contained a both FXa and FVa) had rapid coagulant activity across all prey plasmas. This pattern aligns with numerous studies linking venom toxicity to natural diet: snakes tend to evolve venoms most effective against their typical prey [52,53,54,55,56,57,58,59,60,61,62,63,64,65,66,67,68,69,70,71,72,73,74,75]. Specialist venom such as that of *P. porphyriacus*, which primarily targets ectothermic prey (frogs or reptiles) in the wild, appear to be finely tuned to the clotting physiology of such prey, having a faster relative effect than on rodent plasma (Figure 1; Supplementary Tables). Such a trade-off suggests evolutionary refinement of toxin activity, presumably at the molecular level of clotting factor recognition, to preferentially disable non-mammalian hemostatic systems. 

Importantly, prior work has shown that these same venoms are still highly coagulopathic in human plasma [45], indicating that specialization has not eliminated their danger to other animals. Rather, the venom of these specialists may have greater potency against ectothermic prey as a byproduct of optimization for prey selectivity, while still being active against endothermic plasmas. From an evolutionary standpoint, this supports the notion that venom phenotypes can become narrow in scope when a snake’s diet is restricted, potentially sacrificing broad-spectrum potency for precision. Such specialization is documented in other venomous snakes as well. For example, certain viperid species possess multiple toxins that differentially target amphibian vs. mammalian vs. avian clotting factors, reflecting adaptive protein variation for different prey [70,101]. Our data reinforces that concept with Australian elapids, demonstrating clear functional divergence in coagulotoxic effect correlated with prey type.

Conversely, species with generalist diets appear to retain versatile venom activity. In our study, FXa-type venoms from snakes that feed on a broad range of prey did not exhibit strong preferences between amphibian, avian, or mammalian plasma (Figure 1). These venoms caused rapid clotting and strong clots across the board, suggesting a more generalized mode of action. Evolutionarily, this makes sense: a snake that regularly preys on mammals, birds, and reptiles would benefit from toxins that are effective in any prey’s bloodstream. In effect, natural selection in generalists may favor retention of a broad-spectrum arsenal, whereas specialists undergo directional selection that enhances efficacy toward one class of prey at the expense of others. It is notable that all the factor Xa-based venoms tested (including those of small, frog-eating elapids) are capable of initiating robust clot formation in human plasma [45]. This underscores that even highly specialized venom components can still cause dangerous coagulation disturbances in humans. However, the relative drop in potency we observed on “non-target” plasma (rodent) for the three specialists likely reflects an evolutionary calculus: if mammals are rarely on the menu, there may be little selective pressure to maintain peak venom effectiveness against mammalian clotting factors. That it may remain under neutral selection pressure, but being subject to evolutionary drift. Such insights highlight how studying venom effects in diverse plasma types (beyond the standard rodent or human assays) reveals adaptive nuances that would otherwise remain hidden. In practical terms, this suggests that traditional LD_50_ tests in mice (or clotting assays in human plasma) might underestimate the true biological potency of a venom toward its natural prey, or conversely, might overestimate its effectiveness on other taxa. Our multi-species plasma experiments provide a clearer window into the functional evolution of these venoms, validating the hypothesis that prey-driven selection has tangible impacts on toxin function.

Speed of action, however, is but one feature. The strength of the formed clot is an independent variable. An intriguing outcome of this evolutionary theme is the intraspecific variation we discovered within the venom of the Eastern Brown Snake (*P. textilis*). While *Pseudonaja* as a group are often considered diet generalists, there are known genetic lineages within this species [97,98], and these lineages have been associated with divergent diets [102]. While all species and populations were very rapid in action, there were significant differences in the nature of the resulting clots. Our results showed that a southern population of *P. textilis* (from South Australia) produced strong, stable clots in all prey plasmas (including rodent), whereas northeastern populations (Queensland) produced weaker, friable clots in rodent plasma despite clotting it just as quickly. This difference in clot quality between the two populations mirrors a documented phylogenetic split in *P. textilis* (a southern “temperate” clade vs. a northern “tropical/subtropical” clade) and is likely rooted in evolutionary adaptations (Figure 3) [97,98]. One plausible explanation is that the South Australian *Pseudonaja*, which are smaller on average and historically faced different prey availability, have secondarily evolved a more generalized or ancestral venom efficacy.

Phylogenetic bracketing suggests that the last common ancestor of *Oxyuranus* and *Pseudonaja* had a procoagulant venom that formed strong clots (Figure 2). Consequently, the “weak clot” trait arose in the *Pseudonaja* lineage last common ancestor, and predominates in extant *Pseudonaja* species. Our finding that the southern *P. textilis* venom bucked this trend implies an evolutionary reversal: under certain selection pressures, the *P. textilis* venom in that region has re-acquired the ability to form strong clots. This could be driven by shifts in diet or predation strategy. For example, if southern *P. textilis* prey on proportionally more reptiles or have lower venom yields, a stronger clot (though slower to form) might improve prey immobilization and retention. Notably, museum dietary studies have reported that northern clade *P. textilis* attain larger sizes and consume more endothermic prey, whereas those from the southern clade are smaller and take more ectothermic prey on average [102].

These ecological differences could translate into divergent optimal venom profiles. The northern snakes may rely on ultra-rapid clotting factor activation to quickly incapacitate robust mammalian prey (even if the resulting clots are flimsy), while southern snakes might benefit from a clot that is more stable, compensating for lower absolute venom output or targeting reptilian physiology. Supporting this idea, prior work found pronounced venom divergences between Queensland and South Australian *P. textilis*: the Queensland venom was more potently procoagulant (and rich in presynaptic neurotoxins), whereas the South Australian venom had a broader mix of toxins including more post-synaptic neurotoxins [103]. They suggested these differences “may be driven by selection for different prey”, reinforcing that even within one snake species, local feeding ecology can steer venom evolution. Our data provides functional evidence of this, revealing that the coagulotoxic phenotype itself (speed and strength of clot induced) has diverged between populations. In evolutionary terms, this exemplifies the remarkable plasticity of venom and the potential for rapid adaptive shifts, even reversals of trait polarity, in response to ecological pressures.

### 3.2. Variations in Procoagulant Activation Mechanisms

A major focus of our study was the distinction between venoms that utilize only factor Xa-like enzymes and those that deliver a complete prothrombinase complex (FXa:FVa) in the venom [43,44,90,92,104,105,106,107,108,109,110,111,112,113,114,115,116,117,118,119,120,121,122,123,124,125,126,127,128,129,130,131]. All snakes tested ultimately activate prothrombin (whether directly or through upstream activation of Factor VII), but the mechanistic nuances led to fascinating differences in clotting outcomes [47]. *Oxyuranu*s and *Pseudonaja* both possess venom clotting factor activators functionally analogous to the mammalian Xa–Va complex, yet our thromboelastography results revealed a clear genus-level dichotomy. *Oxyuranus* venoms induced rapid clot formation coupled with high maximum amplitude (strong clot strength) in every plasma, including human. By contrast, *Pseudonaja* venoms also triggered extremely rapid clotting (often the fastest of all samples), but the clots formed were of significantly lower strength (lower maximum amplitude values), indicating they were weaker and more prone to fragmentation. Clinically, this dichotomy is intriguing: envenomation by either snake can cause fulminant venom-induced consumption coagulopathy, but the quality of the venom-formed fibrin clot might differ.

Our data suggests that in general *Pseudonaja* venom flood the bloodstream with a torrent of thrombin, forming an unstable “flimsy” fibrin clot that is quickly broken down, a scenario consistent with the sudden fibrinogen depletion and incoagulable blood seen in *Pseudonaja* bite victims [1,6,9,10,19]. *Oxyuranus* venoms, on the other hand, seem to form a more robust clot matrix (at least initially), which could translate to a slightly different coagulopathic profile (potentially involving more significant thrombosis before consumption ensues). These differences likely stem from the biochemical properties of the respective clotting factor activators. It has been shown previously that *Pseudonaja* venoms are even more potently procoagulant (shorter clotting times) than taipan venoms, yet are far less dependent on cofactors like calcium and phospholipids for activity, even accelerating in the absence of phospholipid [46]. This suggests that *Pseudonaja* venoms’ prothrombinase operates in a “fast and loose” manner, in that they work quickly and without needing much co-factor assistance, but the trade-off might be the formation of a less structurally robust clot. *Oxyuranus* venoms, by contrast, may assemble a prothrombinase on endogenous cofactors (akin to the host’s clotting complex), producing a well-crosslinked fibrin clot before ultimately consuming the clotting factors. Further biochemical dissection is needed, but it is tempting to hypothesize that differences in venom FXa heavy/light chains or venom factor V homologs between these genera account for the stark contrast in clot stability. Future work comparing the toxin sequences and bioactivity is required to elucidate these aspects.

The evolutionary implication is that even within the narrow functional category of “procoagulant venom”, snakes have arrived at divergent strategies. *Oxyuranus* might favor a strategy that temporarily secures the prey via a solid clot (which could occlude vessels and cause stroke or organ failure), whereas *Pseudonaja* favor an overwhelming blitz of thrombin that rapidly depletes clotting factors, leading to systemic collapse. Each strategy is lethal, but through different pathological routes: one through sturdy clots leading to swift ischemia; the other through uncontrolled coagulation triggering acute disseminated intravascular coagulation and subsequent hemorrhage.

The case of *P. textilis* underscores how fine-tuned these mechanisms can become under natural selection. As discussed above, the South Australian population’s venom deviates from the usual *Pseudonaja* pattern by generating stronger clots. This reversal to a taipan-like trait suggests a change in the molecular composition of the venom’s prothrombin activator. It raises questions about what molecular changes underlie a “strong clot” vs. “weak clot” venom phenotype. Perhaps the southern *P. textilis* venom has a variant of the non-enzymatic factor V-like subunit that affects clot quality through changes in prothroimbin binding leading to variations in three-dimensional orientation of the FXa enzymatic subunit, or there may changes in the FXa enzymes that interact differentially with prothrombin to produce the divergent effects. Future structure-function studies are warranted to pinpoint these differences. By mapping the specific venom proteins (and their isoforms) in each snake and correlating them with clotting behavior, we can begin to reconstruct the molecular evolution of the prothrombinase complex in Australo-Papuan elapids while linking toxin structure to specific pathophysiological functions.

Phylogenetic analyses have indicated that venom factor X (FXa) toxins evolved only once in Australian elapids and then were refined in a stepwise fashion [45,47]. Within that context, the *Oxyuranus* and *Pseudonaj* represent one branch (the “Group C” prothrombin activators with cofactor FVa built-in). The other branch is represented by the Group D activators which are reliant upon host factor Va: *Cryptophis*, *Demansia*, *Hemiaspis*, *Hoplcephalus*, *Notechis*, *Suta*, *Tropidechis*, and *Pseudechis porphyriacus*. Our results add a new layer, showing that within the Group C branch, there is further diversification: not all FXa:FVa venoms are equal, and even closely related snakes can show opposite effects on clot stability. Elucidating the genetic and structural basis for these functional shifts (including the apparent reversion in southern *P. textilis*) will deepen our understanding of venom evolution. It also exemplifies the concept of convergent versus divergent evolution in venom: different lineages might converge on inducing coagulopathy, but the detailed execution (and resulting pathology) can diverge significantly.

### 3.3. Clinical Implications of Coagulotoxic Variation

From a clinical perspective, our study highlights why snakebite coagulopathy can vary widely between species, and even between populations of the same species. All venoms tested were rapidly procoagulant in human plasma, reinforcing that envenomation by any Australian elapid in this group can cause serious venom-induced consumptive coagulopathy. However, the quality and kinetics of the clot induced can influence the clinical presentation of VICC. For example, the *Pseudonaja* venoms produced clots in human plasma that, while fast-forming, were of lower strength (weak, friable clots). In a bite victim, this likely translates to fibrin clots that are rapidly broken down, manifesting as an early depletion of fibrinogen and high D-dimer levels; the classic picture of “incoagulable blood.” Indeed, *Pseudonaja* envenomations in humans are notorious for causing a sudden collapse and an absence of clot formation on clinical coagulation tests shortly after envenomation, consistent with an acute consumption coagulopathy [1,6,9,10,19].

Crucially, studies that have reported case series of coagulopathic *Pseudonaja* envenomations did not partition the results by species or geographic variants within a species (important for *P. textilis*), instead lumping all the cases together as ‘brown snakes’ [1,3,6,9,10,19,22,26,33]. Which may have obscured differences in the effects produced by the southern *P. textilis.* Such records should be re-examined for differences in the clinical coagulopathic effects of the southern clade of *P. textilis* relative to the northern clade of this species and also relative to all other *Pseudonaja* species. As the southern clade of *P. textilis* is the only *Pseudonaja* species in its region (while the northern clade overlaps with multiple *Pseudonaja* species), using the genetic geographical patterns (Figure 3), partitioning by geographic zone into the southern clade versus *Pseudonaja* from all regions is a feasible approach to re-examining these clinical reports to ascertain if there are variations in coagulopathy produced that parallels the venom variation.

*Oxyuranus* bites also cause VICC, but the initial clot may be more robust; clinicians sometimes observe transient hypercoagulability or thrombotic complications in taipan envenomations before defibrination ensues [6,8,24,25,50,132,133,134,135]. Thus, comparisons of southern *P. textilis* versus northern *P. textilis*/all other *Pseudonaja,* with *Oxyuranus* envenomations will provide another point of reference for strong clot versus weak clot progression in clinical pathology. Recognition of these differences is important. It suggests that viscoelastic testing (TEG/ROTEM) in the emergency setting might reveal a different signature for *Oxyuranus* versus some *Pseudonaja* envenomations. For instance, a *Pseudonaja* bite might show a very low maximal clot firmness (if any clot forms at all) whereas an *Oxyuranus* bite could show clot firmness. Such distinctions could potentially aid in early snake identification and tailored treatment, especially in regions where multiple dangerous species overlap. 

Our findings also have implications for antivenom design and usage. Procoagulant toxins are the primary drivers of life-threatening VICC, so antivenoms must effectively neutralize them. However, as the venom mechanisms differ between weak-clot vs. strong-clot *Pseudonaja* venom phenotypes, this logically indicates structural differences between the toxic enzymes, including changes in the catalytic site. If these changes are at key antivenom recognition sites, particularly the catalytic site, then antivenom efficacy may likewise vary. Prior research has shown that current antivenoms can have differential cross-reactivity: for example, an *Oxyuranus*-specific antivenom was found to only weakly neutralize *Pseudonaja* venom effects despite the close genetic relationship between the genera and the highly similar venom composition [46]. This is in line with our observations that suggest, despite venoms from northern and southern *P. textilis* both being prothrombin and Factor VII activators [47], the toxins responsible are not identical. Consequently, if a *Pseudonaja* antivenom is made using only snakes from either the southern or northern clade, then there may be limited cross-reactivity with the other clade. As the geographic source of the venoms used in the production of current human and veterinary antivenoms are unstated, it is unclear whether the antivenoms are from only one clade, equally split between clades, or some random distribution. Therefore, future work is needed to investigate antivenom cross reactivity across *Pseudonaja* species and regional variations within the *P. textilis* species.

Similarly, the unique venom traits of the amphibian/reptile specialist snake *P. porphyriacus* raise the question of whether it is fully covered by existing antivenoms, which are typically raised against a few major species. Encouragingly, one study on Australian elapids noted that tiger snake antivenom (broadly used for various species) did neutralize some of this procoagulant venom, though efficacy varied by species [45]. For instance, it neutralized *P. porphyriacus* and *S. punctata* venom extremely well but was less effective against *Cryptophis* or *Hemiaspis*. Clinicians should be aware that envenomations by non-standard species (e.g., a small fossorial snake that preys on frogs) might respond differently to antivenom, or progress differently in terms of coagulopathy, compared to bites by well-documented species. This underscores that phylogeny alone is not a perfect predictor of cross-neutralization; rapid venom evolution means even related snakes can differ antigenically, a paradigm reinforced by results with snakes from other regions such as *Trimeresurus* pitvipers [136].

Our results add context: the more a venom’s functional profile deviates (due to specialized toxins or unique clotting factor targets), the more we may need to ensure antivenom antibodies specifically cover those components. This is particularly important for *Pseudonaja* venoms. Current human and veterinary antivenoms are made using just *P. textilis* venom, and the geographical composition of the immunizing mixtures is unstated. Further, there have been no rigorous studies undertaken to ascertain the relative neutralization of geographical variations of *P. textilis*, or the relative neutralization of other *Pseudonaja* species.

## 4. Conclusions

This study provides a foundation for future research that bridges evolutionary biology and clinical toxinology. By mapping prey-specific effects, we can better predict the severity and nature of envenomation by various snakes. For example, knowing that a specialist species’ venom is disproportionately less effective in mammalian blood might suggest that bites to humans, while still dangerous, could exhibit slightly slower clotting effects than an equivalent dose from a generalist species. This is a hypothesis that could be tested with in vivo models. The results simultaneously point to limitations of animal models, especially using non-mammalians to predict human effects. In addition, the extremely rapid action of *Oxyuranus* and *Pseudonaja* venoms on human clotting affirms why time is critical in treating such bites. Consequently, antivenom must be administered as soon as possible to halt the cascading coagulopathy. Understanding the evolutionary context of these venoms also aids antivenom development: if a particular toxin type (e.g., FXa-only vs. FXa+FVa) is responsible for pathology, antivenom producers can ensure their immunogens include representatives of each toxin class.

Moving forward, integrating our prey-specific findings with molecular analyses will help identify the exact venom components behind each coagulotoxic effect. This could lead to improved therapeutics, such as enzyme inhibitors targeting snake FXa or novel adjunct treatments to stabilize clots in the short term. The divergent coagulotoxic strategies uncovered in this research not only illuminate the evolutionary pathways of Australia’s notorious snake venoms but also have direct ramifications for managing and treating snakebite victims. By appreciating both the evolutionary fine-tuning and the clinical outcomes of these venoms, we can better tailor our medical responses and deepen our understanding of how nature’s deadliest cocktails came to be.

Overall, our study demonstrates that examining snake venoms through the dual lenses of ecology and medicine yields valuable insights. Venom is not a static trait; it evolves in response to the challenges of subduing prey, and in doing so it generates the complex clinical syndromes we observe in envenomations. The clear prey-linked differences in coagulotoxic efficacy we observed provide compelling evidence of evolutionary adaptation, while simultaneously suggesting certain clinical divergences between snakebite scenarios. These findings encourage a more nuanced approach to both venom research and antivenom strategies, one that accounts for the biological context of venom use as well as its medical impact. Ultimately, unraveling the interplay between a venomous snake’s diet, its venom biochemistry, and the pathophysiology in bite victims will enhance our ability to predict risks and devise interventions, exemplifying the value of integrative toxinology.

## 5. Materials and Methods

### 5.1. Venom Stock Preparation

All venom samples were sourced from the cryogenic collection of the Adaptive Biotoxicology Lab; they were sourced under University of Queensland, Animal Ethics Approval 2021/AE000075 (15 March 2021), and work was approved by UQ Biosafety Committee Approval # IBC/134B/SBS/2015 (14 April 2023). All venom stocks had congruent potency with previous studies using the same stocks [45,46]. Lyophilized venoms were reconstituted by adding 50% glycerol and deionized water, and protein concentration was determined through the use of a Thermo Fisher Scientific NanaDrop 2000 UV-Vis Spectrophotometer (Sydney, NSW, Australia) and then adjusted to produce a 1mg/mL venom stock. The reconstituted venom samples were stored at −20 °C until further use.

### 5.2. Plasma Sample Preparation

All plasma work was conducted under University of Queensland Biosafety Committee Approval # IBC/149B/SBS/2016 (20 September 2023). Animal plasmas were collected under UQ Animal Ethics Committee Approval #2020/AE000324: *Gallus gallus* plasma was pooled from 9 individuals and collected at University of Queensland Gatton Campus; *Rattus* norvegicus plasma samples were provided by Animal Resources Centre, Western Australia and pooled from 25 individuals; *Rhinella marina* plasma was a pooled sample from 35 wild caught individuals. Pooled human plasma was sourced from the Australian Red Cross under UQ Human Ethics Approval #2016000256 (9 May 2024), and Australian Red Cross Research Agreement #16- 04QLD-10 (2 February 2025). Plasmas were stored in a −80 °C freezer and only taken out before immediate use. Prior to use, plasma was placed in a 37 °C water-bath for five minutes until no longer frozen and pipetted out for use on the TEG 5000 Hemostasis System.

### 5.3. Thromboelastography

The Haemonetics TEG 5000 Hemostasis System was used to measure plasma clot strength. Thromboelastography was performed on the venoms against each plasma type. Each cup was pipetted with 72 μL of 0.025 M CaCl_2_ (Stago catalogue # 00367), 72 μL phospholipid (Stago catalogue #00597), 20 μL Owren Koller (OK) buffer (Stago catalogue #00360), and 7 μL venom before 189 ul of respective plasmas were added. Kaolin was used as a positive control. Negative control for all plasmas was 50% dH_2_O:glycerol.

### 5.4. Statistical Analysis of Thromboelastography Results

GraphPad PRISM 8.1.1 (GraphPad Prism Inc., La Jolla, CA, USA) was used for all data plotting and statistical analyses. All assays were N = 3. Raw data is shown in Supplementary Table S1.

### 5.5. Pseudonaja Textilis Organismal Genetics

The phylogenetic program MrBayes [137], downloaded from https://nbisweden.github.io/MrBayes/download.html accessed 3 May 2025, was run using sequences obtained from Genbank that consisted of 766 base-pair mitochondrial sequences consisting of NADH dehydrogenase subunit 4 (ND4) gene (partial cds) and tRNA-His (complete sequences) and tRNA-Ser genes (complete sequences) [97]. Alignment is in the Supplementary Materials. Figure 3 presented the results with a focus on *Pseudonaja textilis*, with other nodes collapsed. The full tree output file, however, is available in the Supplementary Materials. The tree was rooted using the *Oxyuranus* sequences as the outgroups. The nexus block used is available in the Supplementary Material Run File.

Genetic results were related by ecozone occupied by each clade by subsequent mapping in R (version 4.3.3) [138] using the “dplyr” [139], “geodata” [140], “sf” [141], “stars” [142], “tmap” [143], and “raster” [144] packages. Image editing was performed in Adobe Photoshop. R code and data files are available from Github at https://github.com/LachlanBourke/Pseudonaja_location_map. Alignments, run files, tree output files, and localities are in the Supplementary Materials.

## Data Availability

The original contributions presented in this study are included in the article/Supplementary Material. Further inquiries can be directed to the corresponding authors.

## Supplementary Materials

The following supporting information can be downloaded at: https://www.mdpi.com/article/10.3390/toxins17080417/s1. Supplementary File S1: sequences.fasta; Supplementary File S2: MrBayes_run_file.nexus; Supplementary File S3: MrBayes output tree.tre; Supplementary File S4: localities; Supplementary Table S1: raw data; Supplementary Table S2: statistics of Figure 1 and Figure 2 clot strengths.
