# Supplementary material for: X Marks the Clot: Evolutionary and Clinical Implications of Divergences in Procoagulant Australian Elapid Snake Venoms
Source: Toxins (Basel). 2025 Aug 18;17(8):417. doi: 10.3390/toxins17080417 (PMC12390032; doi:10.3390/toxins17080417)
Supplement: Supplementary file 1 [file toxins-17-00417-s001.zip › toxins-3818540 supplementary material/Supplementary File S4 localities.pdf]

Northern clade

DQ098601.1 *P. textilis* Finke Gorge National Park, Northern Territory
DQ098602.1 *P. textilis* Pinjarra Dam, Yumbarra, South Australia
DQ098604.1 *P. textilis* Werta Wert, South Australia
DQ098606.1 *P. textilis* Goyder Lagoon, South Australia
DQ098607.1 *P. textilis* Goyder Lagoon, South Australia
DQ098609.1 *P. textilis* Renmark, South Australia
DQ098612.1 *P. textilis* Innamincka, South Australia
DQ098616.1 *P. textilis* Merauke, Irian Jaya
DQ098617.1 *P. textilis* Merauke, Irian Jaya
DQ098618.1 *P. textilis* Biloela, Queensland
DQ098619.1 *P. textilis* North Queensland
DQ098620.1 *P. textilis* Malanda, Queensland
DQ098621.1 *P. textilis* Charters Towers, Queensland
DQ098624.1 *P. textilis* Narromine, New South Wales
DQ098625.1 *P. textilis* Nyngan, New South Wales
DQ098628.1 *P. textilis* Ballina, New South Wales
DQ098629.1 *P. textilis* Alstonville, New South Wales
DQ098630.1 *P. textilis* Tibooburra, New South Wales
DQ098631.1 *P. textilis* Mootwingee National Park, New South Wales
DQ098636.1 *P. textilis* Mullaley, New South Wales
DQ098637.1 *P. textilis* Bangalow, New South Wales

Southern clade

DQ098599.1 *P. textilis* Melbourne, Victoria
DQ098600.1 *P. textilis* Melbourne, Victoria
DQ098603.1 *P. textilis* Tailem Bend, South Australia
DQ098605.1 *P. textilis* Mannum, South Australia
DQ098608.1 *P. textilis* Nora Creina Bay, South Australia
DQ098610.1 *P. textilis* Port Germein, South Australia
DQ098611.1 *P. textilis* Richman Valley, South Australia
DQ098613.1 *P. textilis* Wemen, Victoria
DQ098614.1 *P. textilis* Hackham, South Australia
DQ098615.1 *P. textilis* Burra, South Australia
DQ098622.1 *P. textilis* Buddigower Nature Reserve, New South Wales
DQ098623.1 *P. textilis* Mandurama, New South Wales
DQ098626.1 *P. textilis* Scotts Head, New South Wales
DQ098627.1 *P. textilis* Mullaley area, New South Wales
DQ098632.1 *P. textilis* Bendemeer Stn., New South Wales
DQ098633.1 *P. textilis* Mullaley, New South Wales
DQ098634.1 *P. textilis* Mullaley, New South Wales
DQ098635.1 *P. textilis* Mullaley, New South Wales
DQ098639.1 *P. textilis* Glen Innes, New South Wales
DQ098642.1 *P. textilis* Griffith, New South Wales
DQ098644.1 *P. textilis* Minlaton, South Australia
